# Time-of-flight neutron radiography for differentiating ice and water during phase transitions in natural porous media

**DOI:** 10.1038/s41598-026-64838-5

**Published:** 2026-08-05

**Authors:** Michael Lombardo, Jongmin Lee, Amelie Fees, Eric Ricardo Carreon Ruiz, Pierre Boillat, Alec Van Herwijnen, Jürg Schweizer, Peter Lehmann

**Affiliations:** 1 WSL Institute for Snow and Avalanche Research SLF, 7260 Davos, Switzerland; 2 PSI Center for Neutron and Muon Sciences, 5232 Villigen PSI, Switzerland; 3 ETH Zurich, Physics of Soils and Terrestrial Ecosystems, 8092 Zurich, Switzerland

**Keywords:** Porous media, Neutron imaging, Time-of-flight, Radiography, Water, Ice, Energy science and technology, Engineering, Materials science, Physics

## Abstract

Measurement of coexistent liquid water and ice distributions is useful for many materials and systems such as fuel cells, concrete, snow, and soil. Energy-resolved neutron imaging is a promising technique for these measurements by exploiting differences in neutron cross-section between ice and water at cold neutron energies. Until now, energy-resolved neutron imaging techniques have only been used with relatively thin and non-natural samples. Given the importance of liquid water and ice distributions in many natural porous materials, we investigated the feasibility of using time-of-flight neutron radiography for two natural porous materials. Two exploratory experiments were performed with a model system of glass beads (simple soil analogue) and a natural snow sample with sample thicknesses of 5 mm. The experiments demonstrated that the relative attenuation could be used to distinguish between liquid and solid water phases as well as to dynamically track the phase transition within these porous materials. The results also demonstrated that significant challenges with respect to the scattering in thick samples, inconsistent thicknesses associated with spatial variations of the porosity, and time-variant structures of the porous matrix remain.

## Introduction

The presence of H_2_O in liquid and solid forms (water and ice) is important for many processes in porous media. For example, in devices such as lithium-ion batteries or polymer electrolyte fuel cells, solidification of water or the electrolyte can degrade performance or even inhibit startup^1–3^. In natural materials such as soil, snow, and rocks, the ice and liquid water content can dramatically affect their thermal^4–7^, dielectric^8–10^, mechanical^11–14^, and hydraulic properties^15–18^. Particularly in materials such as snow and glaciers, liquid water and ice coexist in a highly dynamic, non-equilibrium state which constantly changes the solid and liquid phase distributions and, subsequently, the properties of the porous medium^19–21^. Understanding liquid water transport and ice formation within natural porous materials is therefore important for a number of fields such as hydrology, natural hazards, material science, and engineering.

Neutron imaging is an attractive choice for non-destructively investigating H_2_O distributions and dynamics due to the strong interaction between neutrons and hydrogen. Neutron interactions with atoms are quantified with the so-called neutron cross-section, which represents the probability of interaction. In general, neutrons interact through scattering and absorption. For H_2_O, the total neutron cross-section is dominated by incoherent scattering and depends on the neutron energy and aggregate state (solid vs liquid), particularly at cold neutron energies (*≲* 12 meV or wavelengths greater than 2.6 Å)^22–26^. The energy dependence of the neutron cross-section reflects molecular vibrational and translational dynamics and, in the solid phase, collective lattice excitations that vary with temperature^26,27^.

Energy-resolved neutron imaging methods exploit this crosse-section difference in liquid and solid states by combining measurements at higher and lower neutron energies. The ratio of the neutron attenuation at low and high energy (low energy/high energy), called the relative attenuation (*α*), is used to identify the H_2_O phase and allows for direct measurement of the H_2_O phase without apriori assumptions or information about the sample thickness^2,25,28,29^. The value of *α* is specific to the experimental setup and depends on factors such as the detector, neutron source, neutron energies, wavelength resolution, and image processing. In previous studies, neutron wavelengths above about 6 Å and below 3 Å were frequently used for the low and high energy measurements, respectively, and resulted in differences of *α* between liquid and solid H_2_O between about 1% and 10%^2,24,25,27,28,30^.

Several energy-resolved techniques have utilized the relative attenuation to identify the phase of H_2_O. A dual-spectrum approach utilized a Beryllium filter to acquire low and high energy images and was used to investigate sub-zero startups of small-scale fuel cells^28,31,32^. Alternatively, the time-of-flight (ToF) method enables energy-resolved measurements since the neutron energy is directly related to its arrival time at the detector. Manipulation of the pulse enables optimization of the balance between neutron energy resolution and flux. A so-called high duty cycle ToF method with a beam chopper at a continuous neutron source was used to image freezing and melting processes in polymer electrolyte fuel cells^2,24^ and lithium-ion batteries^25,26^. The beam chopper controls the time intervals during which neutrons are transmitted to the sample. In the case of a high duty cycle, the beam is open for a large fraction of the cycle and the blocking period is relatively short. Finally, a ToF technique at a pulsed neutron source^27^ with a gated image intensifier was used for in-situ imaging of ice formation in automotive polymer electrolyte fuel cells^29,30^. With these methods, H_2_O thicknesses of up to 3 mm were measured in cuvettes and capillaries, while the maximum in-situ H_2_O thickness within fuel cells was reported as 0.3 mm^25,29^. Compared to the materials in these previous studies, natural porous materials present a number of additional challenges. Many natural materials have comparatively large pore and grain sizes, which necessitate thicker samples (on the order of 1 cm) in order to obtain representative results. Increased sample thickness reduces transmission and increases the proportion of scattering to the overall signal. Additionally, the porous structure introduces complexities related to the variable thickness and structural changes of the porous matrix. To our knowledge, these complexities of natural porous media have not yet been investigated with energy-resolved neutron imaging.

Given the importance of liquid water and ice distributions in natural porous materials, we investigated the feasibility of using high duty cycle ToF neutron radiography for natural porous materials. Two exploratory experiments were performed with (i) a model system of glass beads and (ii) a natural snow sample with sample thicknesses of 5 mm (transmission down to about 5%). The model glass bead system was used as a simplified analog for natural porous materials such as soil where the solid matrix does not contain H_2_O and was investigated to determine the baseline difference in the relative attenuation of the ice and water within the pores. The snow sample, on the other hand, was a true natural sample and is the the first investigation (to our knowledge) of energy-resolved neutron imaging applied to a material whose matrix and pore liquid both consisted of H_2_O. The experiments demonstrated that the relative attenuation could be used to distinguish between H_2_O liquid and solid phases as well as to dynamically track the phase transition within these porous materials. Complexities due to the large H_2_O thickness and time-variant pore structure led to large uncertainties, which must be optimized or reduced in future investigations.

## Methods

### Neutron imaging

ToF neutron radiography was performed at the ICON beamline in the Swiss Spallation Neutron Source (SINQ) of the Paul Scherrer Institute (PSI) in Villigen, Switzerland^33^. A continuous neutron beam through a 40 mm pinhole was pulsed by a 300 mm diameter Cd chopper disk (20% duty cycle) rotating at 88 Hz, for which the corresponding wavelength ranges between 1.1 and 7.9 Å^25^. The microchannel plate (MCP) detector with a timepix chip-based sensor was used to capture neutrons^34^. The resulting field-of-view was 28 mm × 28 mm with a 55 $\mathrm {\mu }$m pixel size. Over the period of 1 minute, 109 averaged ToF datasets (frames) were recorded during 11.36 ms pulses (4 times per revolution, 88 Hz). Vertical strips of perfect neutron absorbers (^10^B_4_C “black bodies”) were placed in front of the chamber and were used for the scattering correction described below. Glass columns filled with glass beads or snow were located within a climatic chamber in the beam^35^. Due to differences in the detection efficiency of the MCP detector plates, only one side of the field of view was used for the analysis.

### Sample preparation and experiment execution

**Fig. 1:**
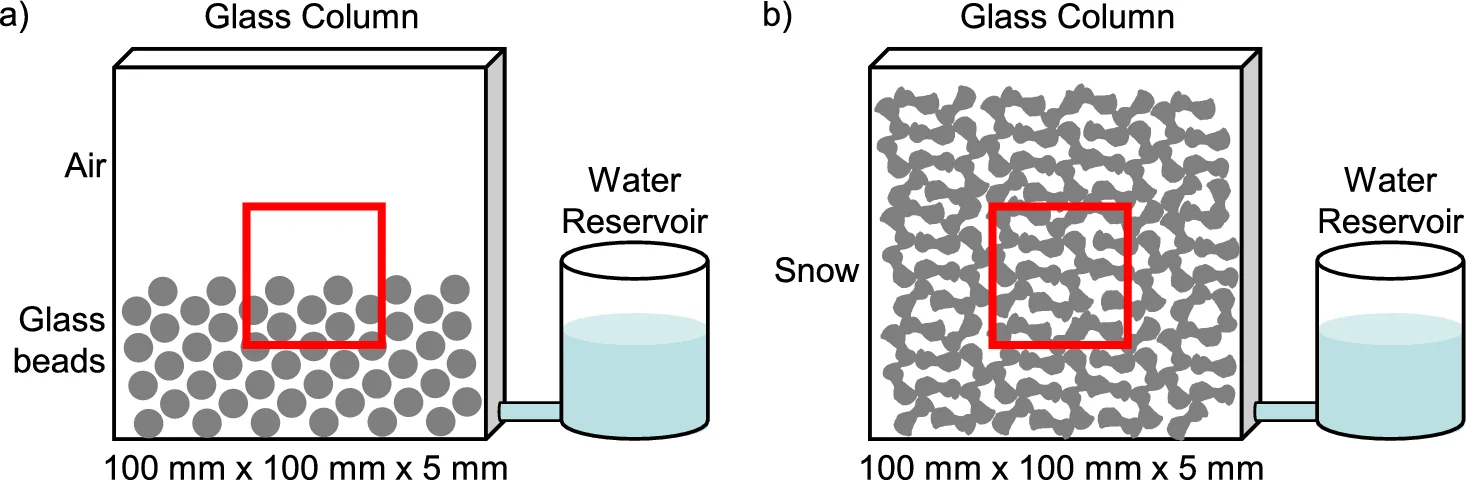
A schematic of (**a**) the model system with glass beads and (**b**) the natural snow sample. The field of view of the detector is outlined by the red boxes (shown orthogonal to the beam direction). The schematic is not to scale.

**Fig. 2:**
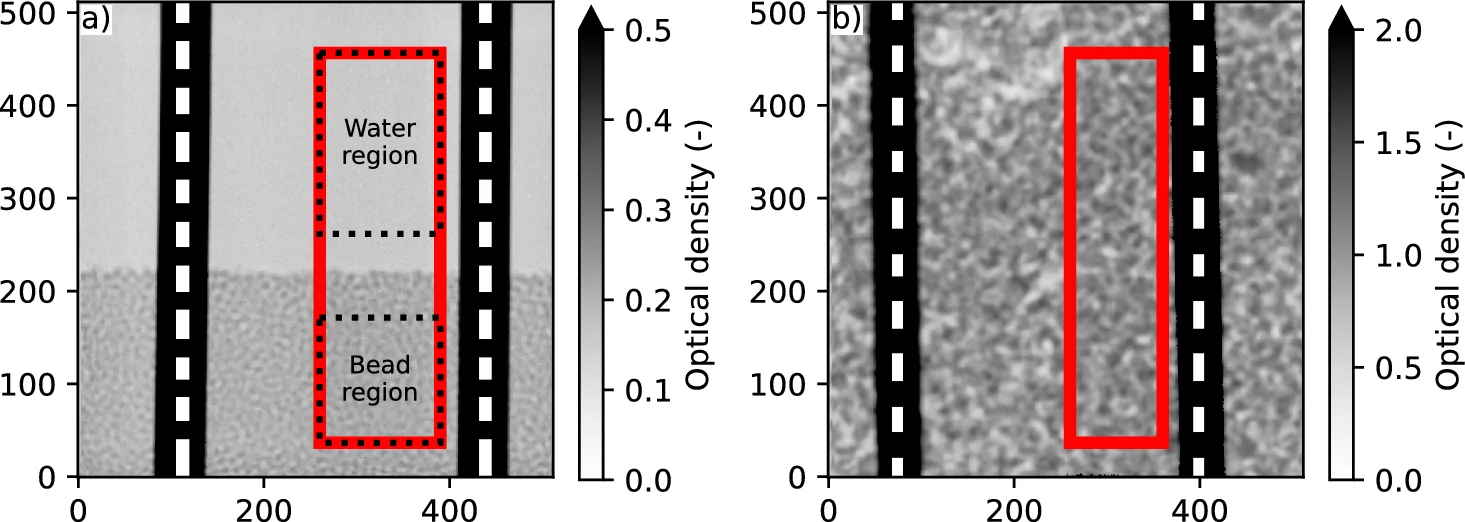
Images of the field of view for (**a**) the column with glass beads in a dry state and (**b**) the column with snow (air-filled pores). Due to differences in the detection efficiency of the detector plates, only one side of the field of view was used for the analysis and is outlined by the red box. For the model glass bead system in (**a**), the specific water and bead regions are outlined with black dashes. The black body strips are shown as the thick vertical black bars and the location of the virtual black bodies that were used for the scattering correction are the white rectangles in the middle of the black body strips. The axes units are pixels. The pixel values are given in optical density which is proportional to attenuation.

One float glass column (100 mm *×* 100 mm *×* 5 mm inner dimensions with 4 mm wall thickness; Glas Würsch GmbH, Zurich, Switzerland) was partially filled with silicone glass beads (1.0–1.5 mm diameter; Sigmund Lindner GmbH, Warmensteinach, Germany) at room temperature (Fig. 1a and Fig. 2a). The column was placed in the climatic chamber and connected to a water reservoir located outside the chamber. After images of the dry column were taken, a valve was opened to let water flow into and fill the column. The temperature of the chamber was set to +1.5 °C and imaging was performed continuously from this point onward, beginning with a set of reference images of the wet column. The chamber temperature was then reduced to −4.5 °C for approximately 4.5 hours to freeze the sample, after which the temperature was increased to +10 °C to remelt the ice.

A similar approach was used for a column with natural snow collected in Davos, Switzerland (Figs. 1b and 2b). The snow consisted of melt forms^36^ with a grain diameter of 1–2 mm as determined with a crystal grid and a density of 482 kg m^−3^ determined with a density cutter (prior to packing into the column). The density within the column may deviate from this value. The column (identical dimensions as described above) and snow were cooled to approximately −15 °C for filling and then transferred to the climatic chamber (−2 °C) for the dry images. This column was also connected to a water reservoir and the column was subsequently filled with water from an ice bath at 0 °C. Continuous imaging was initiated and began with wet snow reference images. After imaging the wet snow (0 °C), the sample was melted at +10 °C. Images at +1.5 °C were taken after the sample had melted to obtain a liquid water reference image.

### Image processing

Image processing was executed with the NeuRED framework and included 3-dimensional filtering, adjustments for the dark current (detector noise when the beam is off), open beam correction, scattering background correction, and adjustment for fluctuations of the incoming neutron beam^37^. Due to the thickness of the samples used here, scattering was expected to contribute significantly to the total measured signal. Therefore, the scattering correction warrants a brief explanation. The scattering correction uses a grid of black bodies (perfect neutron absorbers with zero transmission) to measure the scattered neutron signal behind a grid of black bodies^38^. The full 2D scattering image is then generated by interpolating between these measured points. For our experiments, vertical strips of black bodies were used instead of a grid to improve the vertical resolution from the existing grid with 25 mm spacing. Therefore, virtual black blodies (white rectangles Fig. 2) were generated along the strips and the scattering images were interpolated based on these virtual black bodies. This method has been previously used for relatively thick (1 cm) wet snow samples and was validated using a stepped wedge resulting in an attenuation coefficient of water of 3.6 (+/−0.02) cm^−1^ which matched the reference value of 3.6 cm^−1^^20^.

In addition to the standard neutron radiography processing, the following steps were performed to account for specific features of ToF neutron imaging: correction for pulse overlap (i.e. fast neutrons from a subsequent pulse overlapping with slow neutrons from the preceding pulse)^39^, stack averaging, and wavelength selection (2–3 Å and 6–7 Å for high and low energy images, respectively).

Neutron radiography is based on the Beer-Lambert law

1 $$\begin{aligned} \begin{aligned} I = I_{\textrm{0}}e^{-\mu \delta } \end{aligned} \end{aligned}$$

where *I* is the measured intensity, $I_{\textrm{0}}$ is the incoming intensity before interaction with the sample, *μ* is the attenuation coefficient of the sample, and *δ* is the path length. The parameters *μ* and *δ* are integrated over the sample (and sample components). The attenuation coefficient (*μ*) is related to the neutron cross-section and number density of the sample components^25^.

**Fig. 3:**
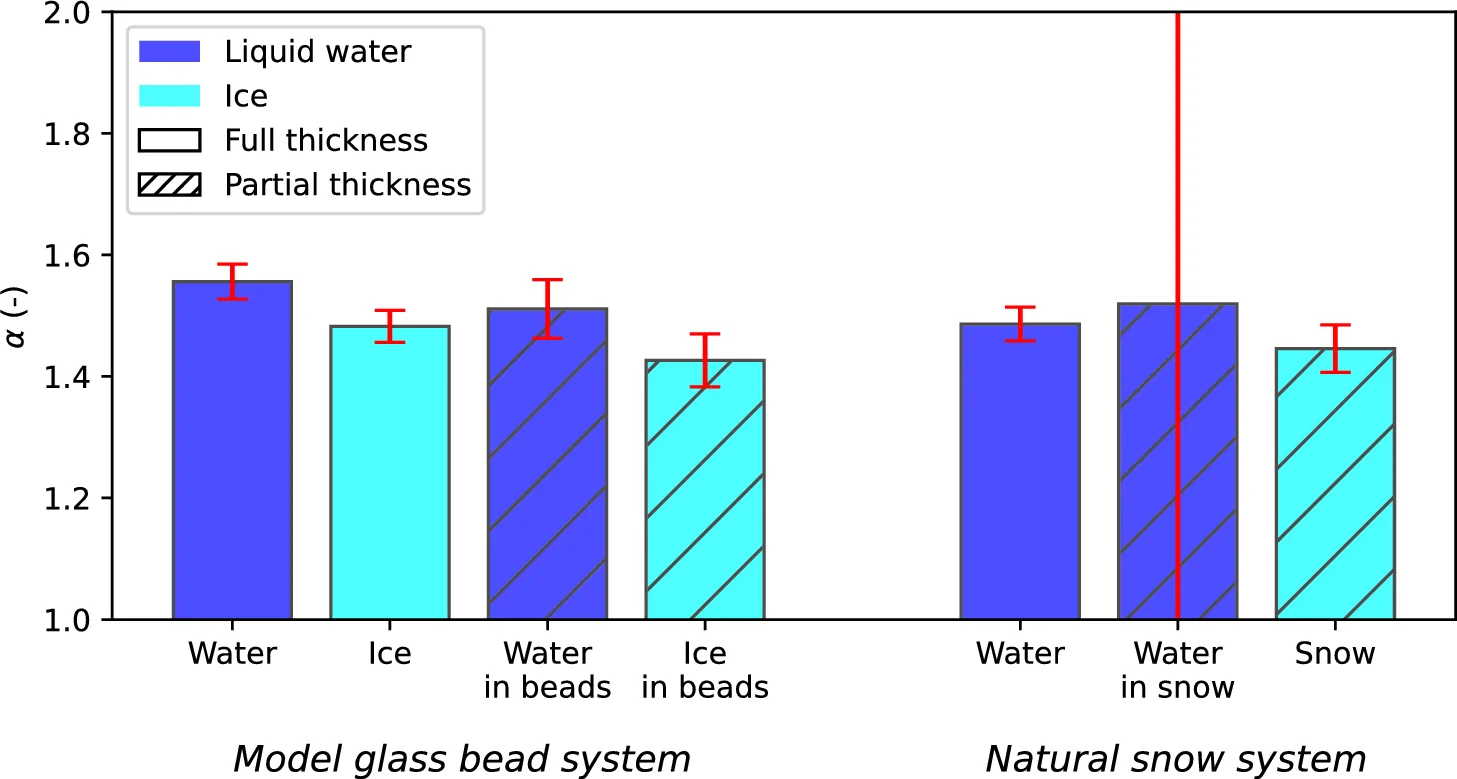
A comparison of the relative attenuation (*α*) of the liquid water (darker blue) and ice (lighter blue) in both experiments. The error bars represent one standard deviation of the pixel values within the region of interest as shown in Fig. 2. The cross hatching indicates when the measured value corresponds to a thickness less than the full 5 mm column thickness. For the model glass bead system values, the reference image is the dry column with beads. For the natural snow system, the reference image for the water and snow values is an empty dry column while the reference image for the water in snow is the dry snow image. Thus, the water in snow value is a partial thickness measurement of only the liquid water phase within the ice structure. These values were obtained by averaging over approximately 1 hour of measurements. The large standard deviation for water in snow was caused by enclosed air bubbles and time-variant heterogeneities in the snow phase.

By selecting an appropriate reference image for $I_{\textrm{0}}$, the unitless optical density (OD) can be defined as

2 $$\begin{aligned} \begin{aligned} OD = -\ln \left( \frac{I}{I_{\textrm{ref}}}\right) \end{aligned} \end{aligned}$$

where *OD* is the optical density, *I* is the measured image with the aforementioned corrections, and $I_{\textrm{ref}}$ is the relevant reference image (also corrected)^24^. Different reference images were used to obtain different values. For the model glass bead system, optical density values in this manuscript were calculated using the dry column (with glass beads) as the reference image. For the column with natural snow, a dry and empty column was used as the reference image in Fig. 5 and the snow and water values in Fig. 3. The dry snow image was used to obtain the relative attention (*α*) value for the liquid water within the snow pores (denoted as “water in snow”) in Fig. 3. The selection of these reference images $I_{\textrm{ref}}$ takes into account contributions from non-sample components such as the glass column walls and the climate chamber.

The optical density was calculated for both the low and high energy images, which were then used to calculate the relative attenuation (*α*) as

3 $$\begin{aligned} \begin{aligned} \alpha = \frac{OD_{\textrm{LE}}}{OD_{\textrm{HE}}} \end{aligned} \end{aligned}$$

where $OD_{\textrm{LE}}$ and $OD_{\textrm{HE}}$ are the optical densities calculated with Eq. 2 for the low energy and high energy images, respectively^24,28^. As stated above, only one side of the field of view was used for the analyses due to differences in the detection efficiency of the detector plates (Fig. 2).

## Results and discussion

### Phase identification

The experiments showed that the relative attenuation (*α*) can be used to differentiate between liquid and solid phases in the porous materials (Fig. 3). In the water region of the model glass bead system (full 5 mm H_2_O thickness), there was a clear difference in *α* between liquid water (1.56 +/- 0.03) and ice (1.48 +/- 0.03). In the bead region (partial H_2_O thickness), the difference between liquid water (1.51 +/- 0.05) and ice (1.43 +/- 0.04) was similar, but the absolute values were lower.

**Fig. 4:**
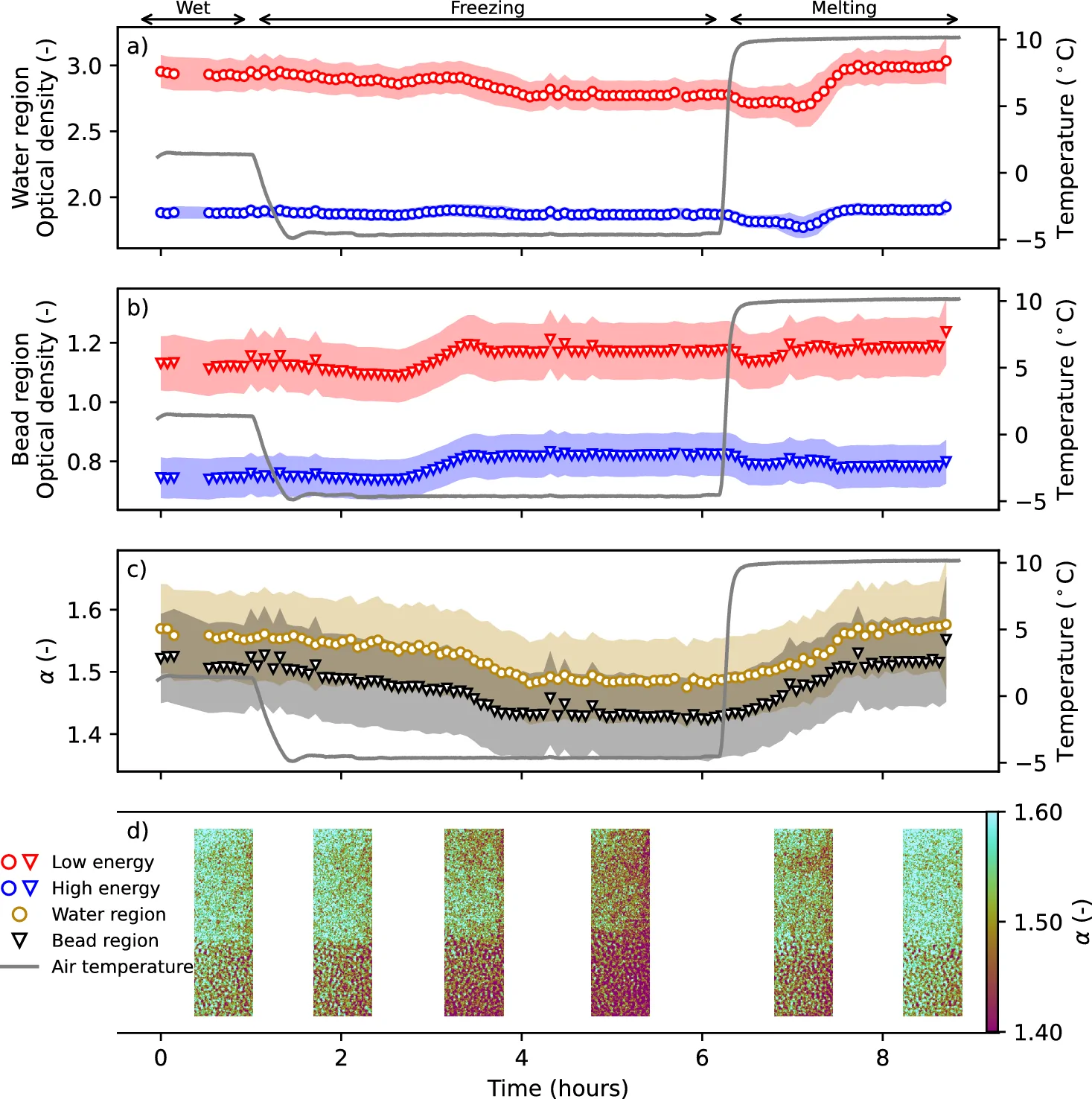
A timeseries of the freezing and melting phase transitions of the model glass bead system shown with the optical density (**a** and **b**) and relative attenuation (**c** and **d**). The water (**a**) and bead (**b**) regions correspond to the regions shown in Fig. 2a. The relative attenuation (*α*) is shown in (**c**) for water and bead region and selected images are shown in (**d**) with the beads visible in the lower half of each image. The expansion of the bead region during freezing is also visible. The images in (**d**) were smoothed with a gaussian filter with a sigma of 0.5. The labels with arrows on the top of the plot show the different stages and phase transitions. The gray line is the measured air temperature of the chamber and is read on the right y-axis. The shaded regions represent one standard deviation of the pixel values within the region of interest as shown in Fig. 2.

**Fig. 5:**
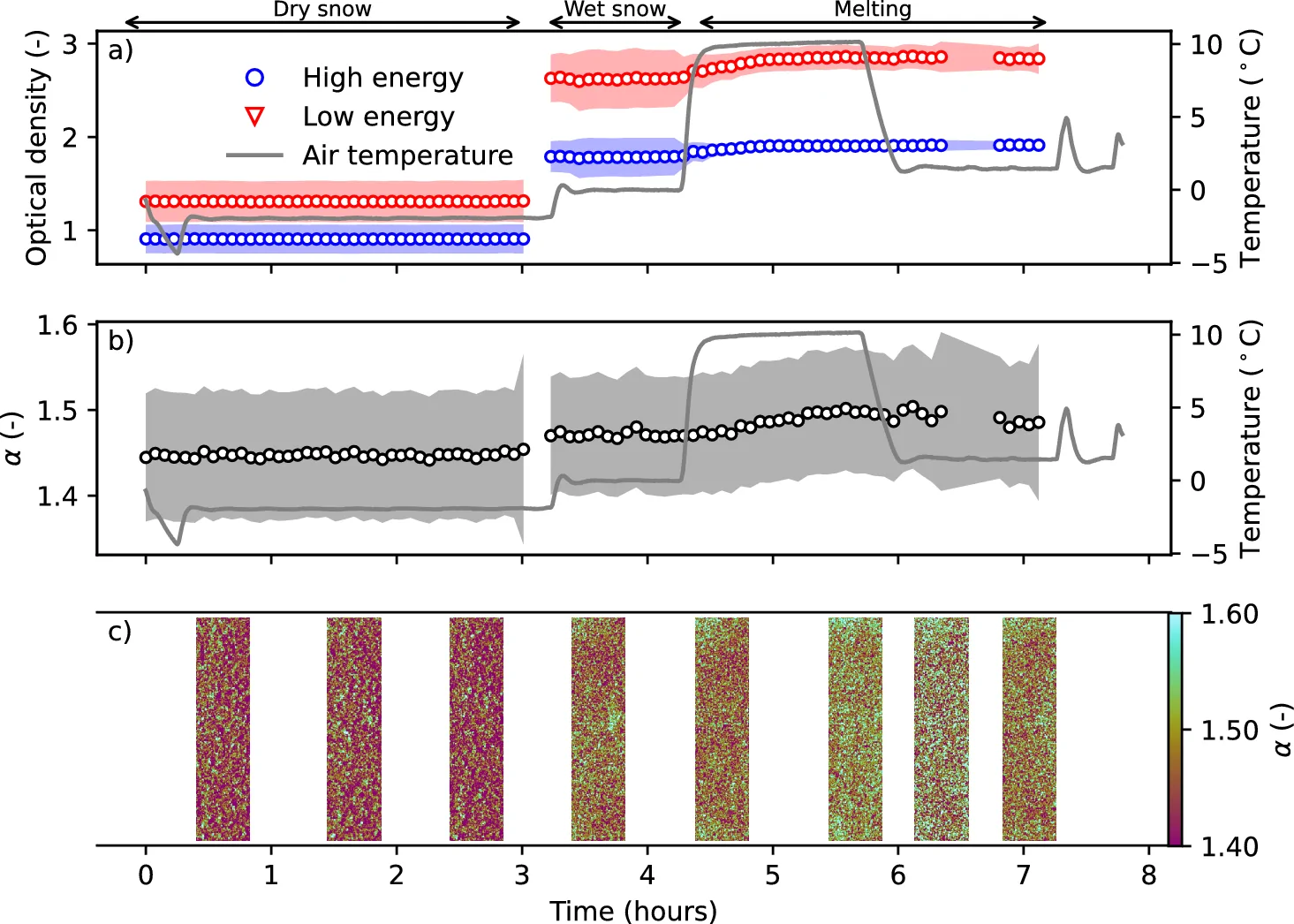
A timeseries of the dry snow, wet snow, and water phases of the natural snow sample shown with the optical density (**a**) and relative attenuation (**b** and **c**). Selected images of the relative attenuation (*α*) are shown in (**c**) and correspond to the region shown in Fig. 2b. The images in (**c**) were smoothed with a gaussian filter with a sigma of 0.5. The labels with arrows on the top of the plot show the different stages. The dry snow was filled with water during the break in measurements around 3.5 hours. The gray line is the measured air temperature of the chamber and is read on the right y-axis. The shaded regions represent one standard deviation of the pixel values within the region of interest as shown in Fig. 2.

For the natural snow sample, it is difficult to make meaningful comparisons between the phases. First, there are huge error bars on the “water in snow” value (1.52 +/- 0.95) and second, the liquid water within the snow is located within the pore space of the dry snow, so the total H_2_O thickness is also not the same between the air-filled and water-filled pore configurations (see discussion of thickness dependence below). Accordingly, our analysis of the natural snow sample focuses on the changes observed in Fig. 5, where we see that the dry snow has the lowest *α* value (1.45 +/- 0.04), followed by wet snow (1.47 +/- 0.05), and finally the melted sample (1.49 +/- 0.03). This trend is consistent with expectations, but the relatively large error bars indicate significant uncertainty (see discussion of errors below).

While the results indicate that the relative attenuation (*α*) can be used to differentiate between liquid and solid phases in porous materials, *α* appears to depend on the sample thickness (i.e. the amount of H_2_O) which is inconsistent with theory^24,25,28^. This has been observed in previous studies as well and has remained largely unexplained^2,28,30^. These previous studies showed that comparison of *α* values between sample regions can be highly uncertain (particularly with different H_2_O thicknesses), but that changes in *α* within a particular region of sample are much more reliable^2,24,30^. Consistent with these previous studies, our results show that changes in *α* can be used for phase identification within a particular sample region, but not necessarily between regions (here, between the water and bead regions)^2,28,30^. This also applies to comparisons of results between experiments (here, model glass bead vs natural snow), which has been a challenge in other studies as well^2,28,30^. A detailed discussion of errors and limitations is provided below.

### Phase transitions

The experiments showed that dynamic freezing and melting phase transitions were captured by the ToF imaging (Fig. 4 and 5).

#### Model glass bead system

In the model glass bead system, the phase transitions were captured through changes in both the optical density and relative attenuation (*α*) (Fig. 4). Cooling in the wet state (0–2 hours in Fig. 4) was accompanied by a slight decrease in both the optical density (high and low energy) and *α*, which was attributed to the temperature dependence of the cross-sections^2,25,26^.

The freezing transition itself (around 3 hours Fig. 4a–c) exhibited an increase in both the low and high energy optical density (within the bead region it looks like an “overshoot”) and a more subtle slope change in *α*. This is likely due to the release of latent heat during freezing which temporarily increased the temperature ahead of the freezing front^2,23,30^. This increase in optical density and *α* during freezing has been observed in some other studies and is likely a function of the sample thickness, temporal resolution, and cooling rate^2,28,30^. As expected, in the frozen state (around 4–6 hours in Fig. 4), we observed (in comparison to the wet state) no change in the high energy optical density and reductions in the low energy optical density and *α* within the water region. The increase in low and high energy optical density for the bead region (Fig. 4b) is induced by the volume expansion of the water during freezing which reduced the packing density of the beads, as seen in Fig. 4d. However, since *α* is relative, its value still decreases within the bead region during freezing (black triangles in Fig. 4c and Fig. 4d). Thus, the expansion of the bead region did not inhibit tracking of this phase transition. It should be noted that the difference in cross-section between ice and liquid water discussed here includes the density difference (about 10%) and therefore does not require any explicit correction.

The melting and warming transition (6–8 hours in Fig. 4) is more difficult to interpret, because (a) in the water region, a layer of beads was pushed to the liquid surface when water was added to the column. This layer subsequently fell back down during melting, and (b) in the bead region, the beads compacted again. These factors obfuscate possible temperature effects^2,30^. Additionally, previous investigations have shown that the latent heat flux during freezing was significantly faster than the flux during melting, so there may not be an analogous effect for melting^30^. However, the final wet state at the end of the experiment showed that the optical density and *α* values returned to their original values with slight offsets to their starting values, which are attributed to the difference in temperature between the initial and final states (+1 °C vs +10 °C)^25^.

We also observed the offset between *α* values in the water and bead regions as described above. This offset remained relatively constant throughout the experiment and supports the conclusion that *α* may be used for local dynamics, but should be cautiously used for drawing global conclusions^2^. Resolving or better understanding this apparent thickness dependence is crucial for future application of this method to samples where the H_2_O thickness is not known apriori. In this experiment, changes in the relative attenuation and low-energy optical density tracked similarly during the phase transitions (aside from the increase in optical density during freezing). As such, both the relative attenuation and low-energy optical density indicated the H_2_O phase. However, the ultimate goal is to apply the method to samples where the H_2_O thickness is unknown, in which case only the relative attenuation would provide phase information^24,25^.

#### Natural snow sample

Similar to the model glass bead system, changes to the H_2_O phase in the natural snow system were also captured through changes in optical density and relative attenuation (*α*) (Fig. 5). The addition of water to the snow around 3 hours showed an immediate increase in optical density and *α*. Melting beginning around 4.5 hours resulted in a continuous increase in optical density and *α*, which is a combination of the phase transition and increase in temperature of the resulting water. After the temperature was reduced from +10 °C to +1 °C, a slight decrease in the values is observed as expected. The changes in *α* are clearly visible in Fig. 5c and indicate that dynamic phase differentiation is still possible. Unlike in the model glass bead system, no effects from latent heat were observed, which is likely due to the relatively poor signal-to-noise ratio compared to the model system.

### Errors and limitations

Due to the complexity of the measurement method and samples, the errors reported within this manuscript are a function of multiple factors and originate from the measurement method as well as physical properties of the samples. One factor is that the statistical error of the measurements increases with increasing optical density (i.e. lower transmission and therefore fewer detector counts). This is clearly visible when comparing the high and low energy optical densities in Fig. 4a and Fig. 5a and can be remedied by either increasing exposure time (at the expense of temporal resolution) or by reducing the amount of H_2_O. For many natural samples such as soils or snow, samples thinner than 5 mm become unrepresentative of their natural behavior in the field and may limit the value of the results. Thus, this technique may be most suited for investigating systems and processes where the amount of H_2_O is naturally low. For example, the liquid water content of snow is typically below 10% by volume^40,41^, which would significantly increase transmission compared to the fully saturated state measured in this study. Another option, depending on the sample, is to incorporate D_2_O which has a much smaller cross-section and would therefore increase transmission^42^. For snow, an H_2_O ice matrix with D_2_O as the pore fluid is an interesting combination for studying water flow. When considering the use of D_2_O, the difference in melting point (+3.8 °C for D_2_O vs +0 °C for H_2_O) is important.

Another factor is the natural variation in sample (and thus H_2_O) thickness due to the porous structures within the bead region and snow. This is evident when comparing the water and bead regions in Fig. 4a and b, when the snow structure melts in Fig. 5a, and in the difference in errors between the water and bead regions in Fig. 3. The effect is particularly visible for the “water in snow” sample in Fig. 3 for which the standard deviation is almost as large as the value itself (1.52 +/- 0.95). This is a result of the compounding effects of using the dry snow as the reference image and the fact that bubbles were trapped within the snow structure when water was introduced which added large variations in optical density. As seen in Fig. 5, the errors are significantly smaller when the dry column was used as the reference image. Additionally, because the snow structure itself contributes significantly to the attenuation of the sample (compared to the minor contribution of the glass beads), small changes or movements to the snow structure could lead to large changes in attenuation. The effects due to pore structure likely decreases when the pore or grain size is significantly smaller than the sample thickness. However, the inherent spatial variations in H_2_O distribution and morphology between control and in-situ samples have been proposed as a contributing factor to observed discrepancies in *α* and is certainly applicable to both samples in this study^28^. Other minor effects stemming from the sample structure such as refraction at the solid/liquid/air interfaces were negligible due to the relatively small coherent scattering cross-section of hydrogen^43^.

Other potential sources of error come from the measurement method such as the scattering correction and beam sweep. Scattering has been shown to have a large effect on the value of *α*, particularly when transmission is low^2^. The transmission values of our samples were about 13.5% and 5.0% for the high and low energy bands at full thickness, respectively (optical densities of 3.0 and 2.0 in Fig. 4a), which is similar to the “low transmission” configurations in other studies^2^. Therefore, this suggests that a bias in the scattering correction could account for the offset in *α* values between the two regions. The likely culprit of such a bias may be the relatively (compared to vertical) poor horizontal resolution of the black bodies. Including horizontal black bodies in future experiments would improve the scattering correction and should be compared to a reference sample with known water thickness. Beam sweep is an effect caused by the “sweep” of the chopper through the neutron beam and causes 2D spatial variations within the field-of-view of the relative attenuation (*α*)^2^. The effect is due to slight changes in the time-of-flight as the chopper sweeps over the beam (and therefore the field of view), since the time delay between the trigger and the actual passing of the chopper is slightly different for the left and right side of the sample. This likely contributed to the uncertainties in these experiments and should be corrected for in future measurements^30^. However, because beam sweep should predominantly cause horizontal discrepancies, this effect cannot be the cause of the differences between the vertically offset water and bead regions shown above.

Finally, in addition to the aforementioned errors and biases, comparisons between experiments are further complicated by hysteresis in *α*, which is caused by the phase transition history of a sample^25^. Hysteresis effects are applicable to our results because the model glass bead and natural snow systems did not undergo the same phase transitions and we do in fact struggle to compare values between the two experiments (Fig. 3). Furthermore, in the model glass bead system, the temperature of the chamber for the liquid and solid reference images was never the same (+1.5 °C and −4.5 °C, respectively), so the calculated *α* values also include variations due to the temperature dependence of *α*^25^.

Due to the complexity of these sources of error, a quantitative investigation of the contributions from each effect was not possible. Additional experiments are needed to do this. However, because the phase identification of the full thickness (water) region of the model glass bead system is statistically significant, we believe the results from the dynamic phase tracking (Fig. 4 and 5 still demonstrate the feasibility of this technique. Future experiments can be designed to further understand and reduce the magnitude of the uncertainties.

## Conclusions

Two exploratory ToF neutron radiography experiments were performed on a model glass bead system (soil analog) and natural snow system. The experiments showed that both the liquid and solid H_2_O phases as well as the phase transition could be monitored with the relative attenuation (*α*). Contrary to theory, we observed an apparent dependence of the relative attenuation on the H_2_O thickness. We attribute this to the sample thickness itself, which was significantly larger (5 mm H_2_O) here compared to previous studies, and suspect that it may be related to scattering effects. Due to this thickness dependence, the relative attenuation was shown to be applicable for phase distinction within regions of constant thickness, while comparisons between regions of differing thickness and between samples were not reliable. Several factors should be considered when planning future experiments: low transmission (with 5 mm of H_2_O) leading to high statistical noise, uncertainties related to changes of the porous structure (e.g. bead region expansion during freezing), importance of the scattering correction, hysteresis in *α*, spatial variations due to beam sweep caused by the chopper, and complexities arising from variable sample thickness. Due to these issues, we suspect that this technique may be most suited for natural materials and processes with little H_2_O such as water flow in relatively dry soils, materials with low porosity such as cement, or with the selective incorporation of D_2_O^14,42^. The method also shows promise for 2D detection of processes such as preferential flow channels in soil and snow^16,44^. In any case, while ToF neutron imaging has been used to investigate phase transition of H_2_O, the experimental setups are not yet fully optimized and future experiments need to carefully consider the aforementioned factors.

## Data Availability

The data generated and used in this study are available from the corresponding author upon reasonable request.
